# The effect of PM10 on allergy symptoms in allergic rhinitis patients during spring season

**DOI:** 10.1186/1939-4551-8-S1-A35

**Published:** 2015-04-08

**Authors:** Joo Hyun Jung

**Affiliations:** 1 Gachoon University, Gil Hospital, South Korea

## Background

PM10 (particulate matter less than 10 μm) is known as a major air pollutant component that affects allergy symptoms. We have studied the effects of PM10 on allergy symptoms in allergic rhinitis patients during the spring season.

## Methods

We have reviewed allergic symptoms score changes in 108 allergic patients and in 47 healthy controls by evaluating their 120-day symptom diariesfrom February to May 2012. At the same time, the pollen counts and PM10 concentration were also assessed by the city environmental center. We have compared the symptom scores before and 2 days after the PM10 concentration was elevated over 100 μg/m^3^. Additionally, we have also investigated long-term, 120-day observations.

## Results

The PM10 concentration during the 120-days was less than 150 μg/m^3^. There were no significant correlations between the PM10 concentration change and allergic symptom scoresor drug usage. Allergic symptomswere significantly correlated, however, with pollen counts and out-door activity times (*P*<0.001).

## Conclusions

This study demonstrates that PM10 concentrations(less than 150 μg/m^3^)did not influenceallergy symptoms in allergic rhinitis patients during the ASD season in 2012 year.

